# Isolation of Highly Pathogenic Avian Influenza A(H5N1) Virus from Cat Urine after Raw Milk Ingestion, United States

**DOI:** 10.3201/eid3108.250309

**Published:** 2025-08

**Authors:** Elisha A. Frye, Mohammed Nooruzzaman, Brittany Cronk, Melissa Laverack, Pablo Sebastian Britto de Oliveira, Leonardo C. Caserta, Manigandan Lejeune, Diego G. Diel

**Affiliations:** Cornell University College of Veterinary Medicine, Animal Health Diagnostic Center, Ithaca, New York, USA

**Keywords:** influenza, H5N1, highly pathogenic avian influenza, cats, viruses, zoonoses, respiratory infections, raw milk, United States

## Abstract

In 2024, 3 domestic cats in California, USA consumed raw milk contaminated with highly pathogenic avian influenza A(H5N1) virus. Fever and neurologic signs developed; 2 cats died. The surviving cat’s urine tested positive for H5N1 virus by reverse transcription PCR. Raw dairy products pose a risk to both animal and human health.

Highly pathogenic avian influenza (HPAI) H5N1 virus infects felids, causing neurologic signs and death (*1*–*6*). In March 2024, H5N1 clade 2.3.4.4b genotype B3.13 was first detected in milk from dairy cattle, and large numbers of farm cats were reported dead or disappearing from affected farms (*1*,*2*). Urine has been reported as a suitable sample for detecting H5N1 in live cats (*7*). Here, we describe infection of domestic cats with HPAI H5N1 virus after ingesting commercial raw milk, including clinical signs and outcome of infection in affected animals, and report isolation of H5N1 virus from the urine of a surviving cat.

## The Cases

An owner of 4 indoor-only cats living in southern California, USA, purchased 3 individual gallons of raw milk from 2 health food stores on November 17, 20, and 23, 2024; he fed the milk to his cats. Three of the cats consumed the raw milk through November 25. The milk lot numbers were included in a recall by the California Department of Public Health after multiple products tested positive for HPAI H5N1 virus (*8*). All cats were current on rabies vaccination. 

On November 25, two of the cats, a 14-year-old male neutered brown tabby domestic short hair cat (cat 1) and a 4-year-old male neutered black and white domestic short hair cat (cat 2), had lethargy, anorexia, and fever develop. Cat 1 died on November 28 while hospitalized at an emergency veterinary clinic, and cat 2 died on November 30. No testing for H5N1 virus was performed on either cat. 

Cat 3, a 5-year-old male neutered 5.9-kg tabby cat, displayed similar clinical signs and was hospitalized on December 1. His fever was 40.1°C (104.2°F). He received supportive care, including intravenous fluids, antibiotics (doxycycline and ampicillin/sulbactam), antinausea medication (maropitant citrate), an appetite stimulant (mirtazapine), and a nonsteroidal anti-inflammatory drug (robenacoxib). He was discharged on December 2 but was then admitted to a second emergency veterinary clinic on December 3.

At admission to the second clinic, the cat was anorexic and dull and had hind limb ataxia and paresis. His temperature was 39.7°C (103.5°F) during the night of December 3. A complete blood count, chemistry panel, abdominal ultrasound, and 3 view thoracic radiographs were performed. The only abnormal finding was consolidation of the left ventral lung field. A urinalysis was not performed. The cat was administered similar supportive care with the addition of oseltamivir phosphate (12 mg/12 h for 8 d). The cat also received pradofloxacin and gabapentin orally, as well as nasogastric tube feeding. 

While cat 3 was at the second clinic, the cat owner contacted the San Bernardino County Department of Public Health (SBCDPH) regarding concerns for HPAI virus infection and was advised to seek medical or veterinary care if he or his cats had clinical signs develop. The owner’s fourth cat, a 5-year-old female spayed domestic shorthair, did not drink raw milk and did not develop clinical signs. The emergency clinic gave the owner no recommendations regarding cat 4. SBCDPH was not aware of cat 4 and did not recommend further testing or monitoring based on lack of clinical signs in any human contacts.

On December 4, cat 3 progressed to recumbency and lacked a menace response in the left eye. Passive range-of-motion exercises were initiated. Urine and nasal swab specimens in sterile containers and heparinized whole blood were collected and sent to the Cornell University College of Veterinary Medicine Animal Health Diagnostic Center (AHDC) (Ithaca, NY, USA). On December 5, the cat lacked a menace response in both eyes, but pupillary light and dazzle reflexes were intact bilaterally. He was more alert and was able to stand on the forelimbs but dragged the hind limbs (Video). On December 7, he was discharged to the owner. Over the next few weeks, he used a wheelchair to ambulate and regained the ability to walk and jump on the hind limbs normally. On December 23, he was examined by a veterinary ophthalmologist and had present but weak menace response, pupillary light reflex, and dazzle reflex in the right eye. 

**Video:** Physical signs in cat infected by highly pathogenic avian influenza A(H5N1) virus after consuming contaminated raw milk, United States. Video shows cat 3 displaying hind end paresis, 2 weeks after milk consumption. The cat survived the infection.

At the AHDC, we tested the nasal swab and urine samples for HPAI virus by reverse transcription PCR (RT-PCR) (*9*). Results for the nasal swab sample were negative, but the urine was positive for influenza A (cycle threshold [Ct] of 35.4405) and for avian influenza H5 (Ct 36.706) virus. We froze the urine sample at −80°C (−112°F) and had results confirmed at the National Veterinary Services Laboratory (Ames, Iowa, USA). On December 6, we informed the attending veterinarian, the California State Animal Health Official, and the California Department of Public Health of the RT-PCR results. 

**Figure 1:**
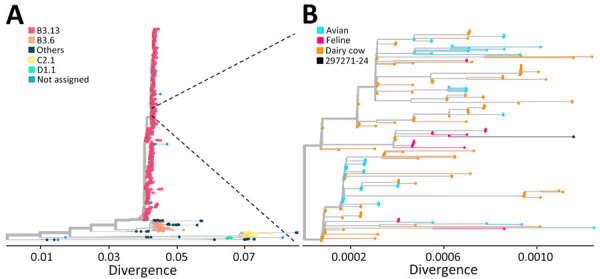
Phylogenetic analysis of complete genome sequences confirming highly pathogenic avian influenza A(H5N1) virus genotype B3.13 isolated from cat urine after raw milk ingestion, United States. A) Tree showing broader phylogeny of H5N1 virus genotypes; B) tree showing closer examination of the virus from the cat urine (sample no. 297271-24) and closely related virus sequences.

On December 21, we performed virus isolation in 10-day old embryonated chicken eggs (ECEs) on the urine sample from cat 3. We inoculated a 1:10 dilution of the urine sample into 10 ECEs via the allantoic cavity route. Four embryos died at 24–48 hours after inoculation. Testing of the allantoic fluids from ECEs that died revealed hemagglutination on turkey red blood cells. The allantoic fluids tested positive for influenza A virus matrix protein by RT-PCR (*9*). In addition, targeted influenza A sequencing confirmed infection with HPAI H5N1 virus genotype B3.13 (Figure 1). We deposited the complete virus sequences into GenBank (accession nos. PV576479–86).

We further performed virus neutralization to detect H5N1 antibodies (*10*). We prepared the heparinized whole blood by centrifuging to isolate the plasma fraction and then tested the plasma. An endpoint neutralizing titer of 1:512 was determined, indicating seroconversion to H5N1 virus after exposure.

**Figure 2:**
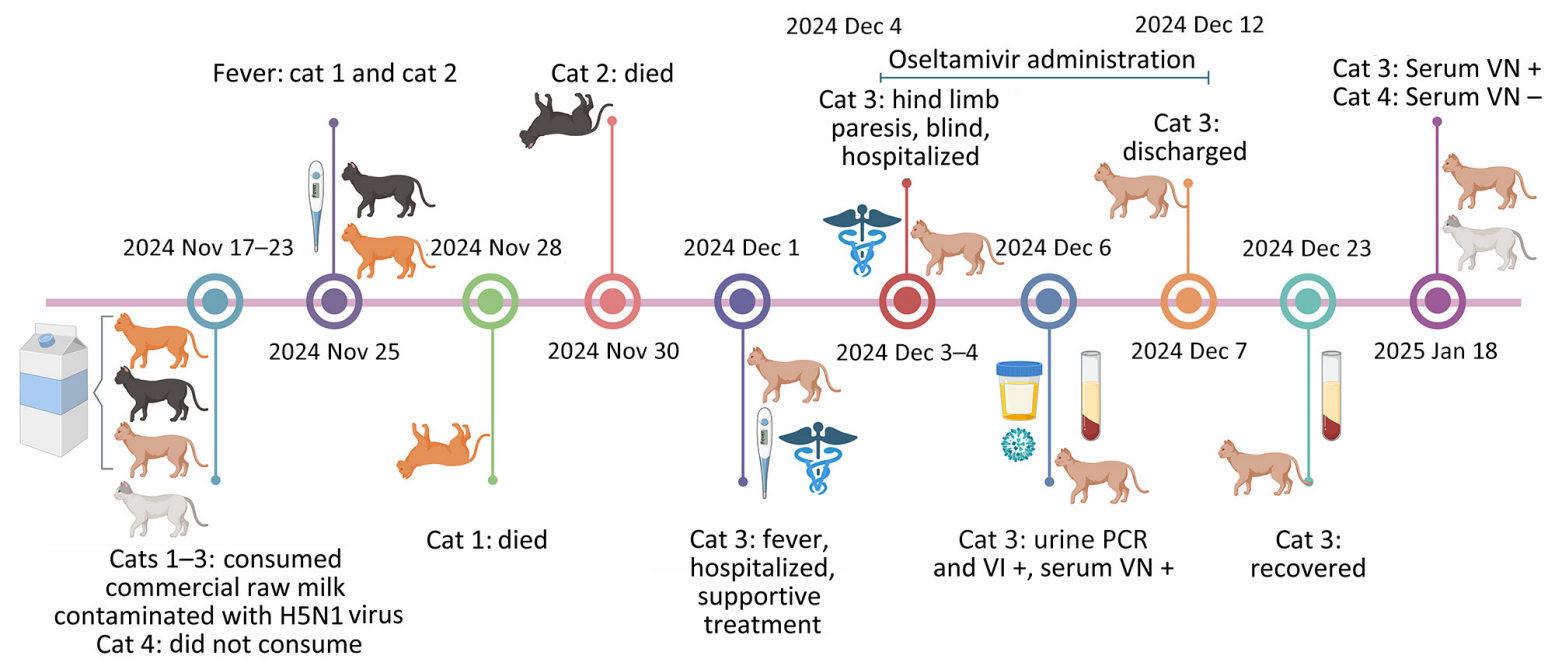
Timeline of events in domestic cats after exposure to highly pathogenic avian influenza A(H5N1) virus through consuming commercial raw milk contaminated with the virus, United States. Cats 1 and 2 died 10–12 days after initial exposure. Cat 3 showed characteristic clinical signs of H5N1 virus infection, was hospitalized and received supportive care including antiviral treatment (oseltamivir), and recovered from infection. Cat 4 did not consume milk and remained healthy throughout the outbreak. Figure created using BioRender (https://www.biorender.com). VI, virus isolation; VN, virus neutralization.

Seven weeks after cat 3 had symptoms develop, the original empty raw milk jugs and serum from cat 3 and cat 4 collected on January 18, 2025, were submitted to the AHDC. Milk residue from 1 of the jugs was positive for H5N1 virus by RT-PCR (Ct 35). However, attempts to isolate virus or sequence from the milk sample were unsuccessful. Cat 3 (clinically affected cat that recovered) had a virus neutralization titer of 1:1,024. Cat 4 was negative for H5N1 antibodies, confirming a lack of exposure (Figure 2).

## Conclusions

This report provides evidence of HPAI H5N1 virus infection in domestic cats after consuming raw milk contaminated with the virus. Two cats died after a disease course characteristic of H5N1 virus infection in felids; however, no diagnostic test confirmed H5N1 virus infection. A third cat had hind limb paresis and blindness develop but recovered after hospitalization and supportive care, which included administration of the antiviral drug oseltamivir. Cat 3 tested positive for H5N1 virus, and a robust H5N1-specific neutralizing antibody titer developed. The raw milk consumed by the cats that had clinical signs of H5N1 virus infection was confirmed positive for HPAI H5N1 virus RNA by RT-PCR. The cat in the household that did not consume raw milk remained seronegative. 

Given the broad circulation of H5N1 virus in dairy cattle in the United States, our results highlight the risk posed by raw dairy products to both animal and human health. Veterinarians examining cats with a history of exposure to wild birds or ingestion of raw poultry or dairy products and acute neurologic signs should have H5N1 infection on their differential diagnosis list.
