# Supplementary figures and images for: Isolation of Highly Pathogenic Avian Influenza A(H5N1) Virus from Cat Urine after Raw Milk Ingestion, United States
Source: Emerg Infect Dis. 2025 Aug;31(8):1636–9. doi: 10.3201/eid3108.250309 (PMC12309769; doi:10.3201/eid3108.250309)

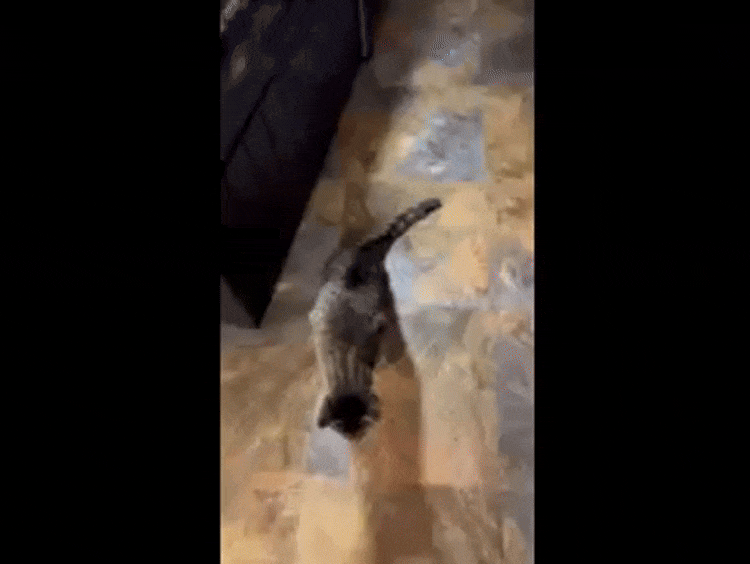

Supplement: Supplementary file 1 [file 25-0309-V.gif]
